# Tissue-on-a-Chip: Microphysiometry With Human 3D Models on Transwell Inserts

**DOI:** 10.3389/fbioe.2020.00760

**Published:** 2020-08-04

**Authors:** Christian Schmidt, Jan Markus, Helena Kandarova, Joachim Wiest

**Affiliations:** ^1^cellasys GmbH, Kronburg, Germany; ^2^MatTek In Vitro Life Science Laboratories, Bratislava, Slovakia; ^3^Centre of Experimental Medicine SAS, Slovak Academy of Sciences, Bratislava, Slovakia; ^4^Institute of Biochemistry and Microbiology, Faculty of Chemical and Food Technology, Slovak University of Technology, Bratislava, Slovakia; ^5^Heinz Nixdorf Chair of Biomedical Electronics, Department of Electrical and Computer Engineering, Technical University of Munich, Munich, Germany

**Keywords:** microphysiometry, transepithelial electrical resistance, label-free monitoring, intestinal model, automated air–liquid interface

## Abstract

Microphysiometry has proved to be a useful tool for monitoring the energy metabolism of living cells and their interactions with other cells. The technique has mainly been used for monitoring two-dimensional (2D) monolayers of cells. Recently, our group showed that it is also possible to monitor the extracellular acidification rate and transepithelial electrical resistance (TEER) of 3D skin constructs in an automated assay maintaining an air–liquid interface (ALI) with a BioChip extended by 3D-printed encapsulation. In this work, we present an optimized multichannel intestine-on-a-chip for monitoring the TEER of the commercially available 3D small intestinal tissue model (EpiIntestinal^TM^ from MatTek). Experiments are performed for 1 day, during which a 60 min cycle is repeated periodically. Each cycle consists of three parts: (1) maintain ALI; (2) application of the measurement medium or test substance; and (3) the rinse cycle. A cytotoxic and barrier-disrupting benchmark chemical (0.2% sodium dodecyl sulfate) was applied after 8 h of initial equilibration. This caused time-dependent reduction of the TEER, which could not be observed with typical cytotoxicity measurement methods. This work represents a proof-of-principle of multichannel time-resolved TEER monitoring of a 3D intestine model using an automated ALI. Reconstructed human tissue combined with the Intelligent Mobile Lab for *In vitro* Diagnostic technology represents a promising research tool for use in toxicology, cellular metabolism studies, and drug absorption research.

## Introduction

Despite well-established tests in the preclinical stage of drug development, unrecognized toxicity is a frequent reason for failure in the clinical stage (Kola and Landis, 2004; Marx et al., 2016). In the study of drug toxicity, it is important to consider absorption effects in the intestine. Preclinical *in vivo* evaluations often rely on mouse or rat models to represent human characteristics (Le Ferrec et al., 2001). However, rodent models cannot reliably predict every aspect of a drug’s effects on humans; therefore, misinterpretations of drug toxicity or resorption can emerge. Thus, the focus has shifted to improving *in vitro* toxicity studies in recent years. The advantages of *in vitro* tests include reproducibility and the ability to separate influencing variables (Ferrick et al., 2008), contributing to better understanding of the mechanisms of toxicity. The limited physiological relevance of two-dimensional (2D) cell cultures and cell lines (Leonard et al., 2010; Ayehunie et al., 2018) and the lack of fully automated analysis for experiments performed in cell culture have been discussed in the literature (Gómez-Sjöberg et al., 2007; Meyvantsson et al., 2008; Li et al., 2013). Recent developments in the field of 3D cell culture address these limitations and demonstrate that new models are needed to increase confidence in the safety and efficacy of new drugs (Fang and Eglen, 2017; Park et al., 2018). Tissue-on-a-chip approaches are implemented to improve assessment of absorption, distribution, metabolism, and excretion (ADME) via *in vitro* models (Madden et al., 2018).

The Caco-2 monolayer culture, derived from a colon (large intestine) carcinoma, is a widely used cell line for *in vitro* assessment of drug absorption and toxicity. It can represent three penetration pathways: transcellular, paracellular, and carrier-based. Unfortunately, its physiological relevance toward small intestinal tissue is limited. It has been described in the literature that it only can accurately predict transcellular permeation (Shah et al., 2006), while other pathways show reduced permeability compared with their *in vivo* counterparts. In addition, the stationary monolayer Caco-2 culture lacks cell–cell and cell–extracellular matrix interactions, so it cannot model the multilayered and complex structure of the human small intestine. To overcome this lack of physiological relevance, new 3D reconstructed human tissue models for absorption analysis have been developed (Li et al., 2013; Ayehunie et al., 2018). The 3D intestinal models are cultured at an air–liquid interface (ALI) and form structures typical of human small intestine tissue. These unique features minimize the morphological drawbacks of submerged 2D *in vitro* cultures (Nossol et al., 2011).

Common approaches for drug exposure analysis using *in vitro* tissue cultures rely on manual and serially performed steps requiring numerous tissue replicates. The variety of methods and measurements also requires manual transfer of cells or tissues into method-specific hardware, which may lead to cell damage and increased variability of results (Blume et al., 2010).

**FIGURE 1:**
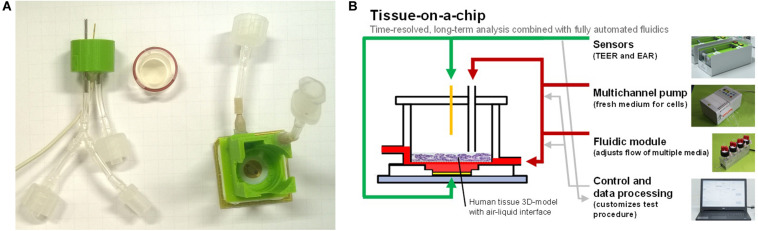
**(A)** BioChip-TEER setup: printed encapsulation with TEER electrode and ALI connectors (left), 3D tissue model insert with sealing ring (middle), and BioChip (24 × 24 mm^2^) with microsensors and printed encapsulation for basolateral fluidic connectors (right). **(B)** Tissue-on-a-chip: multiple sensors, an automated fluidic system for apical and basolateral medium exchange, a control/data processing unit, and a 3D tissue model enable predefined and reproducible experiments.

Time-resolved analysis of the excipient’s effect on cell viability in parallel with its effect on absorption represents an improvement compared with manual, serial performance of absorption and toxicity analysis. Furthermore, with time-resolved data, it is possible to perform both short- and long-term investigations with a single setup. For long-term studies, the manual exchange of media and the addition of reagents must be replaced by an automated system to increase throughput and reproducibility (Kempner and Felder, 2002). In summary, there is a need for systems that allow time-resolved measurements of cell cultures in which multiple parameters can be observed simultaneously and which are extended by an automated fluidic system supporting ALI culture. In this study, such a testing system for a tissue-on-a-chip cell culture with an ALI is presented (Figure 1). The described tissue-on-a-chip setup was constructed by extending an existing BioChip with a 3D-printed encapsulation and was used in a proof-of-principle experiment with a 3D reconstructed human intestinal model (EpiIntestinal^TM^, MatTek *In Vitro* Life Science Laboratories, Bratislava, Slovak Republic).

In this work, we present for the first time a three-channel, fully automated tissue-on-a-chip system that measures transepithelial electrical resistance (TEER), providing further insight into the morphology and barrier properties of the tissue. Notably, the system has an automated ALI in each of the three channels and uses less cell culture medium compared with the previously described one-channel skin-on-a-chip system (Alexander et al., 2018). Therefore, it is now possible to perform three parallel tissue-on-a-chip experiments in one assay, e.g., test substance, control, and blank.

## Materials and Methods

### Human 3D Tissue Model

The experiments were performed using the reconstructed 3D intestinal tissue EpiIntestinal-FT. This human-cell-based 3D model incorporates enterocytes, Paneth cells, M cells, tuft cells, and intestinal stem cells in a differentiated, polarized epithelium. The EpiIntestinal-FT tissue (SMI-100-FT) includes an underlying lamina propria containing normal human intestinal fibroblasts. It is cultured at the ALI to model physiological (luminal) exposure conditions. EpiIntestinal recapitulates different aspects of intestinal function, including barrier function, metabolism, inflammation, and toxic response (Ayehunie et al., 2018).

### Microphysiometric System

The Intelligent Mobile Lab for *In Vitro* Diagnostic (IMOLA-IVD) is a microphysiometric system (Brischwein and Wiest, 2019) extended by an automated fluidic system. The detailed setup of the device was described in a previous report (Weiss et al., 2013). Briefly, it involves BioChips equipped with two interdigitated electrode structure sensors, two electrochemical pH sensors, one amperometrical oxygen sensor, and one temperature sensor. The measurements from the sensors are digitalized and transferred to proprietary software on a computer. The Data Acquisition and Link Application (DALiA) client 3.1 is responsible for data processing and control of the fluidics. The software uses a customizable ON/OFF protocol to control a peristaltic pump and the fluidic system with its bidirectional junction valves. With these tools, it is possible to monitor up to six IMOLA-IVDs in parallel to simultaneously obtain time-resolved insights into, e.g., the effects of a substance plus those of its positive and negative control.

For the maintenance of an ALI with the IMOLA-IVD device, a new encapsulation was developed in Alexander et al. (2018) and refined in this study. The encapsulation was printed with an Ultimaker 3D printer in polylactide and glued onto the BioChip. With this encapsulation, it was possible to have two distinct fluidic lines: an apical and a basolateral line. The apical line could also be used to measure the TEER across a 3D tissue model cultivated on an insert with a semipermeable membrane. The TEER measurements were obtained using one gold wire from the apical side and one gold electrode from the basolateral side. The frequency was 10 kHz, and the applied voltage was 30 mV. The BioChip combined with the encapsulation and the fluidic head for the apical line are depicted in Figure 1.

**FIGURE 2:**
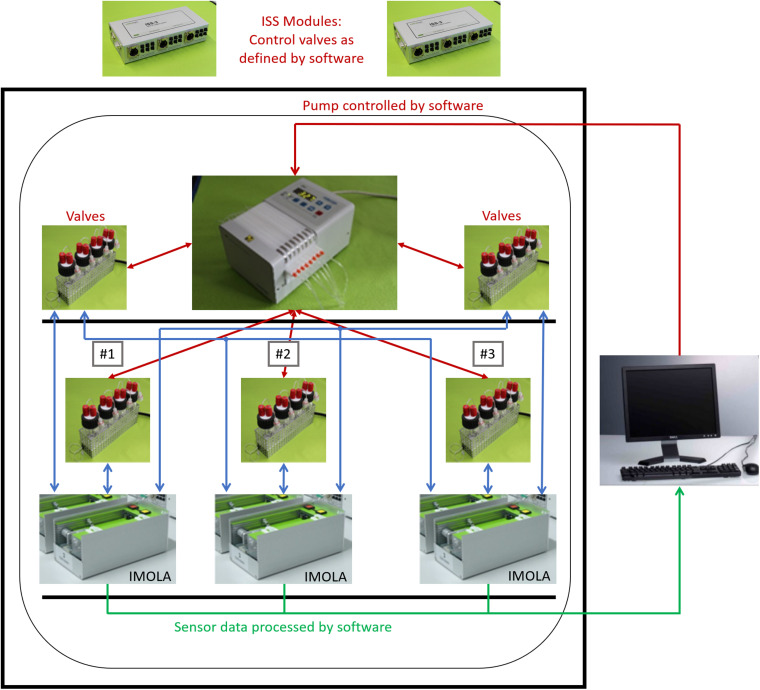
Three-channel tissue-on-a-chip setup. Three IMOLA systems, a peristaltic pump, and fluidic modules are set up inside an incubator to enable measurement at 37°C. Two IMOLA support systems and a PC with proprietary software allow control of the setup.

The complete setup is depicted in Figure 2. The IMOLA support systems (ISS-3) include a power supply for the IMOLA systems, electronics to switch the valves of the fluidic systems, and electronics to record data from bubble detectors. The proprietary DALiA client software application allows control of the measurements and recording of the data. A pump, the fluidic modules with the cell culture media, and the IMOLA systems are mounted inside a commercial incubator to enable measurement at 37°C.

**FIGURE 3:**
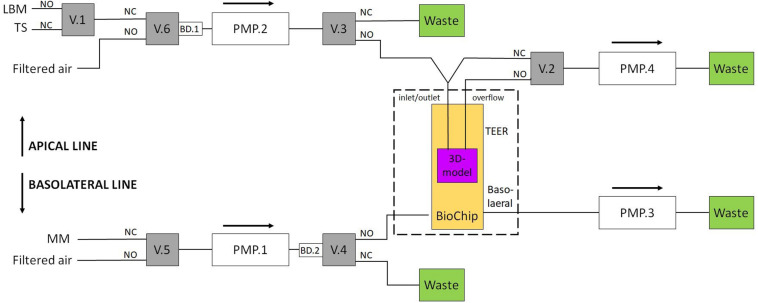
Setup for the two fluidic lines. A TEER measurement liquid or a test substance can be applied via the apical line. Valves 2 and 3 regulate the ALI. For the basolateral line, valve 4 is used to provide new nutrients to the BioChip. Valves 5 and 6 are switched to filtered air when the medium in the other line is pumped (MM, maintenance medium; TS, test substance; LBM, low buffered medium; V.1, valve #1; PMP.2, pump channel #2; BD.1, air bubble detector #1; NC, normally closed; NO, normally open).

Each of the three channels employs two fluidic lines, an apical and a basolateral line, as depicted in Figure 3.

Both fluidic lines can be used to introduce multiple substances to the cells, e.g., culture medium and test substances. In this work, the apical line could apply two different media to the insert: the culture medium and a test substance. During the TEER measurements, the apical side of the tissue was covered with the respective medium to create a conductive connection between the apical and basolateral sides (liquid interface). Otherwise, the apical side was covered with a thin layer (<200 μm) of medium (air interface). The basolateral line periodically provides new nutrients to the basolateral side of the tissue. In this study, the fluidic system was connected as shown in Figure 3. Valve 1 was used to change the apical medium; valve 2 was responsible for filling or emptying the insert’s apical side; valves 3 and 4 were switched to transport the apical and basolateral medium to either the BioChip or the waste container, respectively, and valves 5 and 6 were switched to filtered air when the medium in the other line was pumped. One IMOLA-IVD line uses six valves and four pump channels of a 12-channel peristaltic pump that is controlled in unidirectional mode. The setup presented here used three IMOLA-IVD lines to investigate three tissue inserts in parallel (three BioChips, each with an apical and a basolateral side).

### Assay Preparation for Verification

For verification of the fluidic system setup and measurement procedure, the system was tested with phosphate-buffered saline (PBS) using a shortened protocol. For the apical and basolateral lines, PBS with an osmolarity of 300 ± 10% mOsmol/kg was used. The test substance applied via the apical channel was a PBS liquid of 1000 ± 10% mOsmol/kg.

### Assay Preparation for the EpiIntestinal^TM^ Model

After arrival, tissues were pre-incubated to recover from shipment. They were fed with a 5 ml basolateral medium SMI-100-FT-MM (MatTek *In Vitro* Life Science Laboratories); 200 μl of SMI-100-MM (MatTek *In Vitro* Life Science Laboratories) was added apically to keep the tissues hydrated; and the tissues were incubated for at least 24 h at 37°C and 5% CO_2_.

Before the experiments, the fluidic system was sterilized and pre-filled with media. For sterilization, all tubes and the apical fluidic head were disinfected with 2.5% hypochlorite (Sigma Aldrich, #71696, diluted with double-distilled H_2_O) for 20 min, and the BioChip encapsulation was covered with 70% ethanol for 20 min. Afterward, the media for the basolateral and apical channel were pre-filled into the tubes. An incubator was used to stabilize the temperature of the whole system and the media at 37°C overnight. Finally, the insert containing EpiIntestinal tissue was placed on the sterilized and pre-filled BioChip with the encapsulation. The medium used within the basolateral channel was an unbuffered maintenance medium SMI-100-FT-MM (MatTek *In Vitro* Life Science Laboratories). The apical channel switched between unbuffered DMEM (cellasys GmbH, Kronburg, Germany: SOP-G200-006; see supplement material) and a test substance dissolved in unbuffered DMEM. Sodium dodecyl sulfate (SDS) in concentrations of 0.2 or 2.0% was used as test substance known to disrupt the barrier of the 3D intestinal tissue.

The temporal succession of both fluidic channels was determined in a predefined pump cycle. The TEER measurement cycle consisted of 35 min ALI followed by 10 min TEER measurement and a 15 min rinse cycle. Here, the first ALI period was used for internal pre-filling of the fluidic system. The programmed 1 h cycles were repeated for 24 h. After 8 h, the apical medium was changed to a medium containing 0.2% SDS for one cycle and changed back to an SDS-free medium afterward.

## Results

### Verification of the Measurement Procedure and the Fluidic System

**FIGURE 4:**
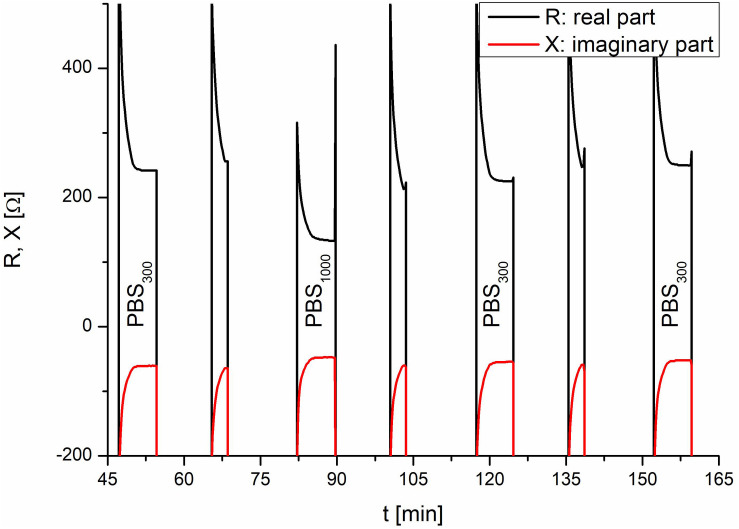
System verification using PBS. In the TEER measurements, the addition of the test substance and the corresponding rinse cycles are visible. The lower TEER values of PBS with an osmolarity of 1000 mOsmol/kg at 80 min correspond to the test liquids and demonstrate the proper functioning of the setup.

Each TEER measurement cycle consisted of a measurement phase and a rinse phase, which were identically constructed for consistency and to guarantee that the effects observed during and after application of the test substance were caused by the test substance. Without this consistency, changes could not be uniquely assigned to the test substance, as other causes would be possible, e.g., a different fluidic flow during the second refill. At the beginning of each TEER measurement, the apical side of the insert was empty and was slowly filled with the respective medium. Once a certain level was reached, the TEER electrode was submerged in the respective medium and was able to measure the resistance. The measurements taken for system verification are shown in Figure 4. The amplitude of the TEER’s real part was 244 Ω at the end of the normal TEER. With the addition of the test substance, which had an increased conductance compared with PBS, the amplitude of the resistance’s real part decreased to 132 Ω. The test substance was diluted by refilling the insert with PBS and then sucked out of the insert a second time. Without this second refill, the test substance would have remained in contact with the cells until the next TEER cycle. As shown in Figure 4, two rinse cycles were required to completely remove the test substance. Taken together, these results demonstrated that the setup of the fluidic system and the sensors of the IMOLA-IVD device were working correctly. Furthermore, it was possible to conclude that the sensors were precise and showed distinct amplitude changes in correspondence to the osmolarity of the applied PBS.

### Control Experiments

**FIGURE 5:**
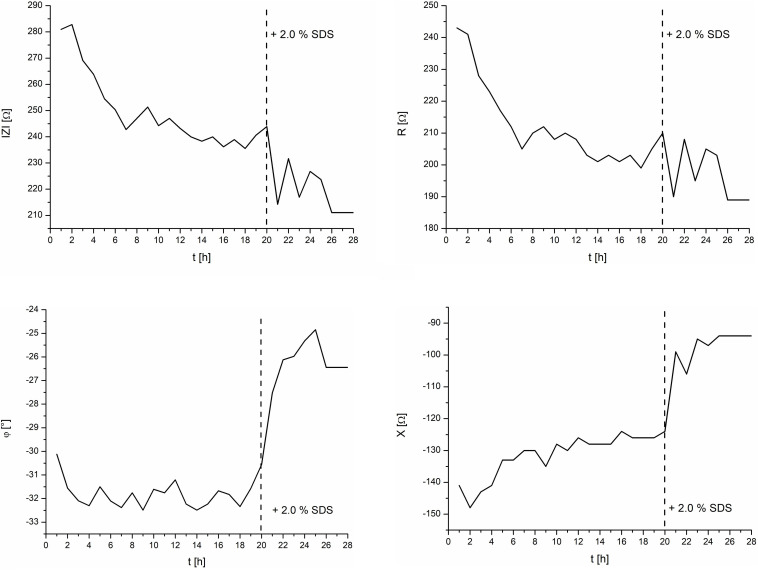
TEER control measurements of the intestine model and the effects of 2.0% SDS. **Left:** magnitude and phase. **Right:** real and imaginary parts.

To validate the setup, a control experiment with negative and positive controls on one IMOLA was implemented. TEER values across an EpiIntestinal tissue were monitored for 20 h (negative control) without the addition of any substance, followed by monitoring from 20 to 28 h under the influence of 2.0% SDS (positive control). The TEER measurements were performed every hour and had the characteristics shown in Figure 4. Thus, during each measurement cycle, TEER values could be described by one mean value approximating the plateaus at the end of the PBS measurements, as depicted in Figure 4. These plateau values were merged to a continuous TEER plot. Figure 5 shows TEER values represented as magnitude and phase (left), and real and imaginary parts (right). These results validate the setup.

TEER values are often displayed as magnitude only. However, to show all measured values, the measured impedance was displayed fully in both common coordinate systems, i.e., in polar coordinates (left) as magnitude and phase, and in Cartesian coordinates (right) as real and imaginary parts. The corresponding mathematical expression is given in Formula 1:

(1) $$Z=R+iX=\left|Z\right|\left(\cos\varphi +\text{isin}\varphi \right)=\left|Z\right|e^{i\varphi }$$

with

Z: Impedance, R: Real part, i: Imaginary unit, X: Imaginary part, |Z|: Magnitude, φ: Phase, e: Euler’s number.

### Tissue-on-a-Chip Model

**FIGURE 6:**
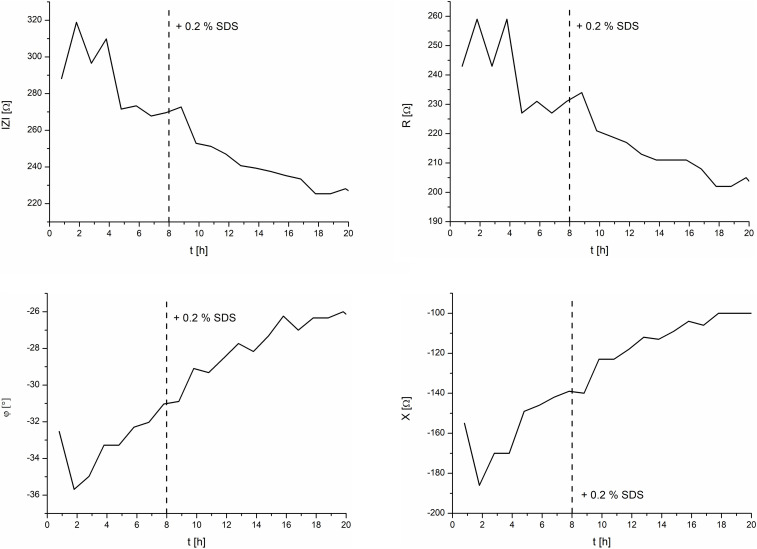
TEER measurements of the intestine model and the effects of 0.2% SDS. **Left:** magnitude and phase. **Right:** real and imaginary parts.

Figure 6 depicts the effect of 0.2% SDS on the EpiIntestinal model. After an initial settling time of about 5 h, the TEER magnitude had a mean value of 270 Ω. Upon exposure of the tissue to the test substance SDS at 8 h, the TEER magnitude spiked slightly and decreased linearly to a final value of 225 Ω at 18 h. It should be noted that SDS does not contribute to the osmolarity of cell culture media. Measurement of the osmolarity (OSMOMAT 030, Gonotec, Berlin, Germany) of the cell culture media with 0, 0.2, and 2.0% SDS resulted in similar osmolarities (300 ± 5% mOsmol/kg).

## Conclusion

Here, we present proof-of-principle experiments using a three-channel microphysiometric system to measure the TEER of a reconstructed intestinal epithelium model (EpiIntestinal^TM^) with an ALI. Parameters are measured non-invasively and in real time, and the system provides automated flow of the fresh nutrients to the tissue on a regular basis. For the TEER measurements, the apical side of the insert was filled. The measurement method and the complete system were successfully tested in a verification experiment with PBS and a control experiment with EpiIntestinal tissues, which were exposed to 2.0% SDS after 20 h. For the tissue-on-a-chip experiment, the procedures were programmed to maintain an ALI, enclosed by TEER measurements. After 8 h, the effect of the test substance (0.2% SDS) was investigated. The results showed a decreasing TEER after the application of SDS. In future work, we propose follow-up experiments with test substances and additional data from microsensors for pH and dissolved oxygen (Wiest et al., 2016).

Overall, the extension of the IMOLA-IVD system with additive manufacturing technology has proven to be a flexible and customizable system for analysis of various cellular models. These include models such as immortalized 2D cell lines, 3D spheroids, and tissue-on-a-chip for the reconstructed human epidermis and intestine. Future work will include investigation and optimization of fluidic cycles (e.g., by increasing the stabilization time before the test substance is added) and the electrode geometry, and collection of spectrometric information (Gilbert et al., 2019; van der Helm et al., 2019), to create a versatile tissue-on-a-chip tool that can also be applied to lung or other mucous cell models.

## Data Availability Statement

The datasets generated for this study are available on request to the corresponding author.

## Author Contributions

All authors listed have made a substantial, direct and intellectual contribution to the work, and approved it for publication.

## Conflict of Interest

JW was the CEO and a shareholder of cellasys GmbH. The remaining authors declare that the research was conducted in the absence of any commercial or financial relationships that could be construed as a potential conflict of interest.
